# Phenotypic heterogeneity is a selected trait in natural yeast populations subject to environmental stress

**DOI:** 10.1111/1462-2920.12243

**Published:** 2013-09-03

**Authors:** Sara L Holland, Tom Reader, Paul S Dyer, Simon V Avery

**Affiliations:** School of Life Sciences, University of NottinghamUniversity Park, Nottingham, NG7 2RD, UK

## Abstract

Populations of genetically uniform microorganisms exhibit phenotypic heterogeneity, where individual cells have varying phenotypes. Such phenotypes include fitness-determining traits. Phenotypic heterogeneity has been linked to increased population-level fitness in laboratory studies, but its adaptive significance for wild microorganisms in the natural environment is unknown. Here, we addressed this by testing heterogeneity in yeast isolates from diverse environmental sites, each polluted with a different principal contaminant, as well as from corresponding control locations. We found that cell-to-cell heterogeneity (in resistance to the appropriate principal pollutant) was prevalent in the wild yeast isolates. Moreover, isolates with the highest heterogeneity were consistently observed in the polluted environments, indicating that heterogeneity is positively related to survival in adverse conditions in the wild. This relationship with survival was stronger than for the property of mean resistance (IC_50_) of an isolate. Therefore, heterogeneity could be the major determinant of microbial survival in adverse conditions. Indeed, growth assays indicated that isolates with high heterogeneities had a significant competitive advantage during stress. Analysis of yeasts after cultivation for ≥ 500 generations additionally showed that high heterogeneity evolved as a heritable trait during stress. The results showed that environmental stress selects for wild microorganisms with high levels of phenotypic heterogeneity.

## Introduction

Individual cells of genetically uniform populations can exhibit marked heterogeneity despite being isogenic. This is evident in effectively any cell phenotype, including virulence of pathogenic organisms (Halliwell *et al*., 2012; Stewart and Cookson, 2012), cell differentiation and reprogramming (Mirouze *et al*., 2011; Buganim *et al*., 2012), and resistance to antibiotics (Balaban *et al*., 2004; Wakamoto *et al*., 2013) and other stressors (Kale and Jazwinski, 1996; Sumner *et al*., 2012; Bishop *et al*., 2007; Smith *et al*., 2007; Levy *et al*., 2012). Studies in recent years have shown that variation in gene expression between such isogenic cells is the principal basis for heterogeneity. These differences in gene expression may have a deterministic basis, particularly for genes regulated by the cell cycle, biological rhythms, growth rate or cell aging, which typically vary across a cell population (Avery, 2006; Carlquist *et al*., 2012; Levy *et al*., 2012; Ryall *et al*., 2012). In addition, the role of stochastic events that culminate in phenotypic diversification has been widely investigated (Raj and van Oudenaarden, 2008). The processes of gene transcription and translation may contribute to such variation or ‘noise’ in gene expression, for example through bursting events that have been described in prokaryotes and eukaryotes (Elowitz *et al*., 2002; Ozbudak *et al*., 2002; Raser and O'Shea, 2004; Blake *et al*., 2006; Cai *et al*., 2006; Carey *et al*., 2013). Large-scale analysis of expression noise in yeast has indicated that proteins that are essential and/or have house-keeping roles are characterized by low expression variation between cells, whereas noise is higher in proteins whose expression may be transiently important, such as stress response genes (Bar-Even *et al*., 2006; Newman *et al*., 2006; Lehner, 2008). Gene promoter sequences that can determine the level of noise in gene expression in prokaryotes and eukaryotes have been identified (Raser and O'Shea, 2004; Blake *et al*., 2006; Freed *et al*., 2008; Li *et al*., 2010; Hornung *et al*., 2012; Silander *et al*., 2012; Carey *et al*., 2013).

Consistent with the apparent evolution of higher levels of expression noise in stress response genes (Newman *et al*., 2006), it has been widely suggested that phenotypic heterogeneity can confer fitness advantages to populations of single-cell organisms. In a similar manner to genotypic diversity (Reed and Frankham, 2003; Markert *et al*., 2010), phenotypic heterogeneity may create subpopulations that are pre-equipped to survive future changes in their environmental niche or other perturbations. As single-cell phenotypes determined by variable gene expression are not heritable, unlike genotypic or prion-based variants (Halfmann *et al*., 2012), phenotypic heterogeneity is predicted to offer particular advantages in dynamic environments subject to intermittent stress. Such predictions have been tested under laboratory conditions with populations of bacterial or yeast cells, with results showing that dynamic phenotypic heterogeneity is especially favoured in rapidly changing systems (Thattai and van Oudenaarden, 2004; Acar *et al*., 2008; Gaal *et al*., 2010). Furthermore, model organisms manipulated to express greater heterogeneity outcompeted less heterogeneous cell populations under varying selective conditions (Blake *et al*., 2006; Smith *et al*., 2007).

One fundamental question that remains unanswered is the extent to which the insights gained above from laboratory investigations reflect what actually happens with populations in the natural environment. This major gap in our knowledge, recognized in recent papers (Ackermann, 2013; Hsieh *et al*., 2013), is important both to assess the significance of phenotypic heterogeneity in nature and because much work on heterogeneity is justified on the basis of its likely importance in natural systems. Here, we set out to address this question by determining and comparing the levels of heterogeneity of wild yeast populations, as representative microorganisms, from stressed (polluted) versus unstressed environmental sites. Our major conclusion is that non-genotypic heterogeneity is a selected trait in natural environments subject to environmental stress and is a key determinant of survival in such adverse conditions.

## Results

### Organisms from the study sites

In order to test the hypothesis that phenotypic heterogeneity is a selected trait in stressed wild environments, yeasts were isolated from unpolluted and polluted locations at three environmental sites, as detailed in the Methods section. To exclude possible species-specific effects, we compared isolates of the same species at polluted and control locations from each site. Accordingly, we collected and analyzed isolates of the most abundant yeast species at each site. *Cryptococcus podzolicus* was the principal yeast isolated at Site 1, from sediments of pools that were either affected or not by copper contamination. Multiple independent non-clonal isolates of *C. podzolicus* were collected from the polluted and control locations, as we corroborated by the random amplification of polymorphic DNA (RAPD) analyses. *Candida sake* was the principal yeast found at Site 2, and multiple independent isolates were obtained from sediments of streams near a lead-mine outflow. Finally, *Sporobolomyces roseus* was the principal yeast isolated at Site 3, from leaf surfaces near a coking plant, which produces airborne sulphur dioxide. As for Sites 1 and 2, all isolates of the species were confirmed to be independent and non-clonal based on RAPD analyses.

### Phenotypic heterogeneity exists in the wild yeast isolates, and differs between those from polluted and unpolluted habitats

**Figure 1:**
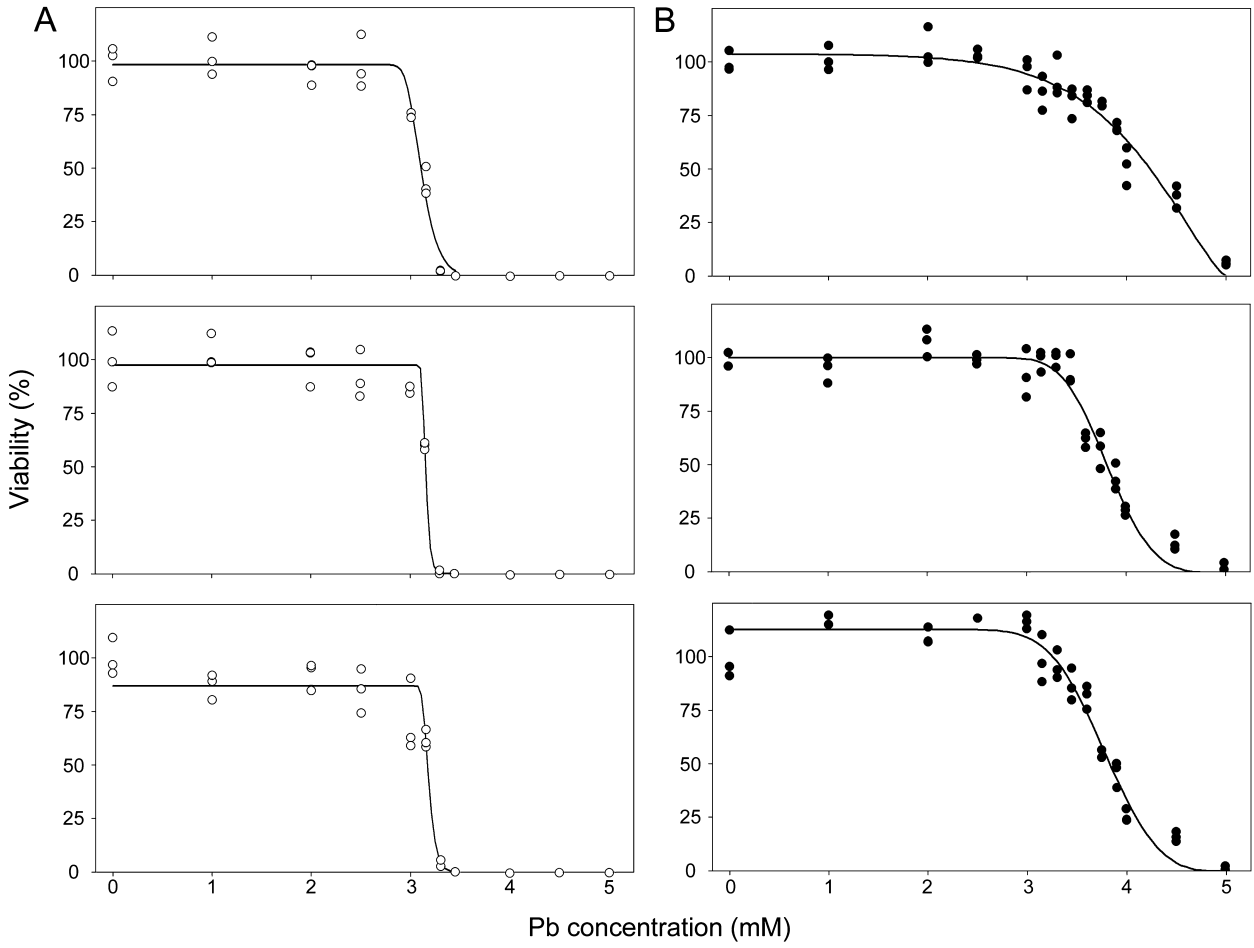
Analysis of heterogeneity. Sample data for *C. sake* isolates from control and Pb-polluted locations. The plots show triplicate independent experiments for each of two example isolates: (A) The least heterogeneous isolate, D1-9, isolated from the control location. (B) The most heterogeneous isolate, D14-7, isolated from the Pb-polluted location. As described in the Methods section, heterogeneity in the relevant stress-resistance phenotype (Pb resistance in this example) was determined from the gradients of the slopes at the point where there was 50% inhibition of colony formation. IC_50_ was determined from the stressor concentration determined to give 50% inhibition of colony formation.

Cell-to-cell heterogeneity is typically measured in relation to a specific phenotype. Here, the phenotype was cellular resistance to the known principal contaminant at the environmental site from which each isolate was obtained. This was appropriate because these contaminants were expected to be primary selective agents at the study sites, and possible selection for increased heterogeneity was the focus of our study. The gradients of dose-response plots (kill curves) provide a convenient measure of cell-to-cell heterogeneity, where the heterogeneity relates to single-cell stress resistances within genetically uniform cultures (Sumner *et al*., 2012; Bishop *et al*., 2007) (Fig. 1). All of the isolates studied here produced a graded decline in colony formation (viability) during culture on agar with increasing concentration of the relevant stressor, similar to those illustrated in Fig. 1. This indicated variation in the stressor doses at which individual cells lost viability, showing that the property of phenotypic heterogeneity is prevalent in wild microbial isolates. Therefore, phenotypic heterogeneity is not restricted to model laboratory organisms. Throughout this study, we routinely corroborated that the differing phenotypes of individual cells were not due to any genetic variation in the relevant culture, as ‘resistant’ or ‘sensitive’ colonies reverted to cultures of cells with mixed phenotypes following subculture in non-selective conditions. That is, the individual-cell phenotypes were transient and not heritable (Fig. S1) (Smith *et al*., 2007). In the following description of results, isolates with high heterogeneity values are those with relatively shallow gradients at the 50% viability (IC_50_) point in dose-response plots, whereas isolates with low heterogeneity are those with relatively steep gradients (Fig. 1).

**Figure 2:**
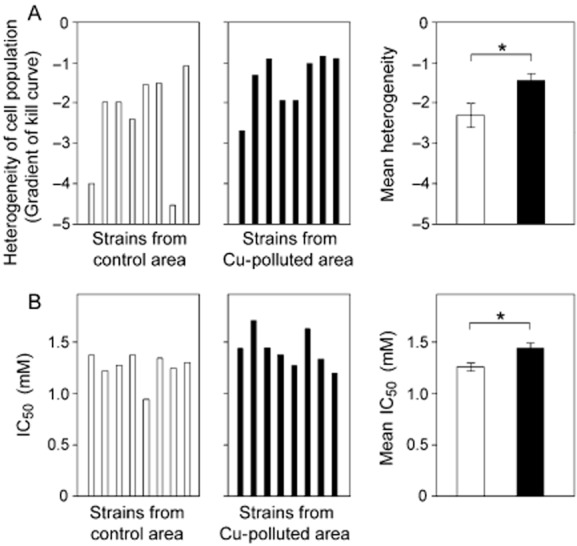
Heterogeneity of *C. podzolicus* isolates near a Cu-polluted mine site. Independent isolates of *C. podzolicus* obtained from control (□) or Cu-polluted (▪) locations at Site 1 were spread plated to MYP agar supplemented with different Cu(NO_3_)_2_ concentrations. Colonies were enumerated after 14 days, and dose-response curves were constructed. Heterogeneity was determined from the gradients of the slopes (A), and IC_50_ from the Cu concentration that inhibited colony formation by 50% (B). Note that a high negative value (e.g. −4) indicates a steep gradient and low heterogeneity, whereas a low negative value (e.g. −1) indicates a shallow gradient and high heterogeneity. Data for each isolate are means from three independent experiments, each performed in triplicate. Isolates are presented in the same order in (A) and (B). The panels on the right show mean values ± SEM of the individual isolate data, which are presented in the left and centre panels. * *P* < 0.05.

Considering Site 1, a total of 16 independent isolates of *C. podzolicus* were collected at the control and Cu-polluted locations at this site. These were subsequently assayed for heterogeneity and IC_50_ following exposure to a range of Cu concentrations (IC_50_ refers to the Cu concentration required to inhibit colony formation by 50% of the cells for each isolate, giving an index of mean or culture-averaged resistance in cell populations). Five of the six most heterogeneous isolates found at the site were from the polluted location, and the least heterogeneous isolates were from the control location (Fig. 2A). This trend was reflected by a ∼ 60% lower mean value for heterogeneity across isolates from the non-polluted versus the polluted location, and the effect was significant (*P* = 0.0416). The mean IC_50_ across isolates from the polluted location was slightly (14%) greater than that of the control isolates (*P* = 0.0165) (Fig. 2B).

**Figure 3:**
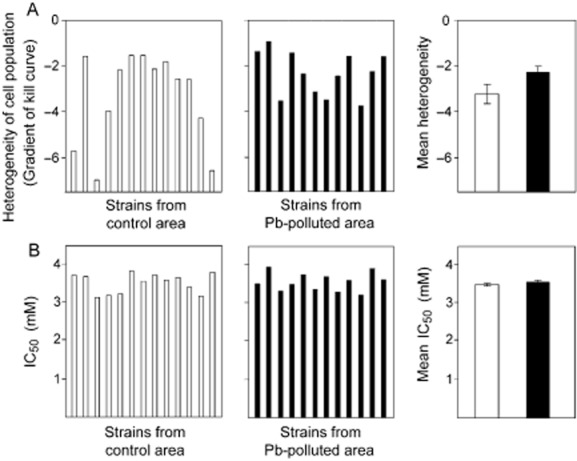
Heterogeneity of *C. sake* isolates near a Pb-polluted mine site. Independent isolates of *C. sake* obtained from control (□) or Pb-polluted (▪) locations at Site 2 were spread plated to MYP agar supplemented with different Pb(NO_3_)_2_ concentrations. Colonies were enumerated after 14 days, and dose-response curves were constructed. Heterogeneity (A) and IC_50_ (B) determinations were as described in the legend to Fig. 2.

Considering Site 2, a total of 25 independent isolates of *C. sake* were collected from the control and Pb-polluted locations at this site. Similar to the trend seen at Site 1, the five least heterogeneous isolates from the control location were all less heterogeneous than any of the isolates from the polluted location (Fig. 3A). Accordingly, the mean heterogeneity was ∼ 42% lower for the control isolates, although the difference was not significant in this case (*P* = 0.0695). In contrast to heterogeneity, the mean IC_50_ across isolates from the polluted location was only marginally (< 2%) greater than that across isolates from the control location. The difference was not significant (*P* = 0.276) (Fig. 3B).

**Figure 4:**
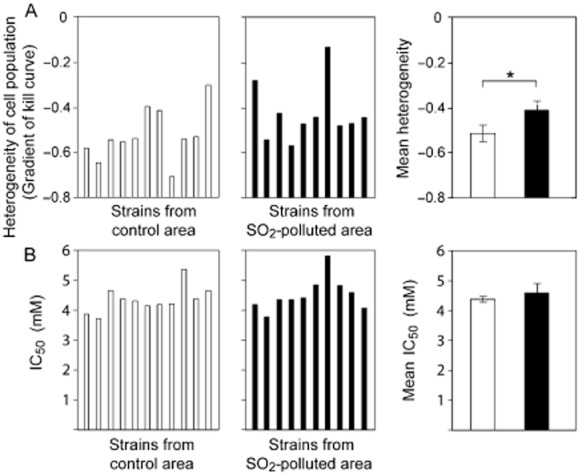
Heterogeneity of *S. roseus* isolates near an SO_2_-polluted coking plant site. Independent isolates of *S. roseus* obtained from control (□) or SO_2_-polluted (▪) locations at Site 3 were spread plated to MYP agar supplemented with different concentrations of sodium metabisulphite (Na_2_S_2_O_5_), which reproduces the toxic action of SO_2_ (Wellburn, 1994). Colonies were enumerated after 14 days, and dose-response curves were constructed. Heterogeneity (A) and IC_50_ (B) determinations were as described in the legend to Fig. 2. * *P* < 0.05.

Yeast populations from the above sites were likely to have been *in situ* in the sediments from which they were isolated for many years. This should encompass hundreds or thousands of cell generations, providing ample opportunity for adaption to pollutants via mutation and selection. To test whether the evidence for increased heterogeneity among pollutant-adapted yeasts may be borne out also in more transient populations, yeasts at Site 3 were sampled from the phylloplane. A total of 21 isolates of *S. roseus* were collected from deciduous tree leaves across the control and polluted locations of Site 3. The results with these isolates were similar to observations from the first two sites: When tested against the principal local pollutant (SO_2_, which acts via the formation of bisulphite), the most heterogeneous isolates were from the polluted location at Site 3, and vice versa (Fig. 4A). Accordingly, the mean heterogeneity across control *S. roseus* isolates was significantly lower than that across isolates from the SO_2_-polluted location (*P* = 0.045). In contrast, the mean IC_50_ did not differ significantly between the two locations (*P* = 0.244) (Fig. 4B).

### Environmental stress promotes evolution of phenotypic heterogeneity

The above data indicated that an increase in phenotypic heterogeneity may be selected as a trait in yeasts from diverse polluted environments. Furthermore, this increase in heterogeneity appeared to be a more strongly selected property than mean stressor resistance (IC_50_). We also concluded that heterogeneity and IC_50_ are not interdependent phenotypes: Heterogeneity and IC_50_ were not significantly correlated across the isolates from five of the six test locations (*P* > 0.05) (the one exception, at the Site 3 polluted location, rested on a single outlying data point; excluding this point gave *P* = 0.922) (Fig. S2).

**Figure 5:**
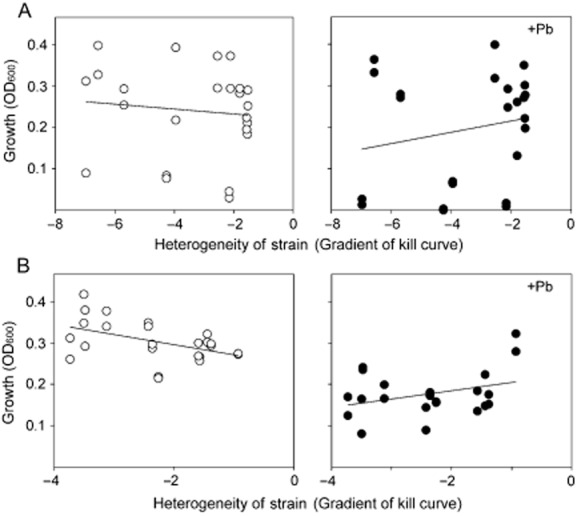
Relationship between heterogeneity and growth in the presence of stressor. *Candida sake* isolates obtained from the control (A) or polluted (B) locations at Site 2, with differing heterogeneities (Fig. 3), were cultured for 3 days with daily subculture to fresh medium in unsupplemented (○) or 15 μM Pb(NO_3_)_2_-supplemented (•) MYP broth. Relative growth of each isolate was determined as OD_600_ after 3 days. Independent biological replicates (×2) for each of the tested isolates are shown for each condition. Plots are model fits from analysis of covariance.

To explain why yeasts with greater heterogeneity predominated in polluted habitats, the first hypothesis we tested was that pre-existing yeasts with high levels of phenotypic heterogeneity might be selected at the expense of low-heterogeneity yeasts after the onset of stress. Isolates from Site 2 were used to test this, as *C. sake* proved particularly amenable to laboratory culture. Isolates with varying heterogeneities from the control (unpolluted) location at Site 2 were cultured in the absence or presence of lead nitrate for approximately three days. The final OD_600_ values were used as measures of each isolate's relative fitness. Isolates with the higher heterogeneity generally appeared to fare slightly better in the presence of Pb, and the reverse trend was apparent in the absence of Pb (Fig. 5A). Indeed, comparison of these relative trends by analysis of covariance indicated that heterogeneity was significantly more advantageous to growth in the presence of Pb compared with the absence of Pb (*P* = 0.026, one-tailed). This relative advantage of heterogeneity was even more significant (*P* = 0.001), when tested in the same way as above, with *C. sake* isolates from the Pb-polluted location (Fig. 5B).

**Figure 6:**
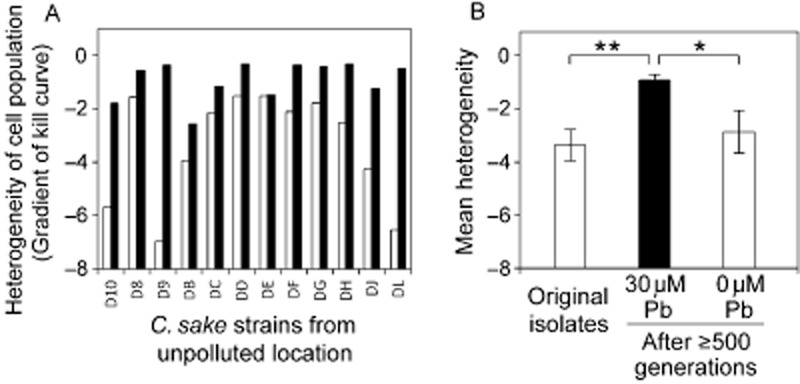
Evolution of heterogeneity during long-term stress.A. *Candida sake* isolates obtained from the control location at Site 2 (Fig. 3), with differing basal heterogeneities (□), were subcultured daily to MYP broth supplemented with 30 μM Pb(NO_3_)_2_ for ≥ 500 generations, before heterogeneity (of Pb resistance) for each was assayed again (▪).B. Mean values ± SEM of the data from (A) and from a parallel control experiment where the isolates were cultured for ≥ 500 generations in unsupplemented MYP broth. The phenotypes evolved after ≥ 500 generations were heritably stable (see main text). * *P* < 0.05, ** *P* < 0.01.

The second hypothesis we tested to help explain why more heterogeneous isolates predominated in polluted habitats was that yeasts evolved increased phenotypic heterogeneity during long-term stress. Gene promoter sequences that affect noise of gene expression have been identified previously, consistent with heterogeneity being an evolvable trait (Raser and O'Shea, 2004; Blake *et al*., 2006; Freed *et al*., 2008; Li *et al*., 2010; Carlquist *et al*., 2012; Hornung *et al*., 2012; Silander *et al*., 2012). To test the hypothesis, *C. sake* isolates from the unpolluted location at Site 2 were cultured for ≥ 500 generations in the presence of 30 μM lead nitrate before being re-examined for heterogeneity. The levels of heterogeneity of each of the 12 tested organisms were increased following this long-term incubation with Pb (Fig. 6A). The mean heterogeneity of the organisms was increased by ∼3.6-fold (*P* = 0.00088, one-tailed) after ≥ 500 generations (Fig. 6B). To rule out the possibility that this change in heterogeneity was related to some parameter(s) of long-term laboratory cultivation other than the presence of Pb, parallel control incubations were performed in which isolates were cultivated for ≥ 500 generations under identical conditions but without Pb. The mean heterogeneity of these organisms did not change significantly during the course of the experiment (*P* = 0.317), and was significantly lower than for the plus-Pb-grown organisms (*P* = 0.029) (Fig. 6B). We had already demonstrated above that heterogeneity and IC_50_ are not interdependent phenotypes in isolates from the wild. Here, the mean IC_50_ values for the organisms before (3.52 μM) and after (3.76 μM) long-term incubation with Pb were not altered significantly (*P* = 0.125). Therefore, the increased heterogeneity after 500 generations arose independently of any significant change in IC_50_.

To substantiate that the increased-heterogeneity phenotypes (Fig. 6) were stable, the heritability of heterogeneity was tested in four of the yeasts that developed the largest heterogeneity differences between plus-Pb and minus-Pb control incubations during the 500-generation experiment. After culturing in the absence of Pb for ∼ 25 generations, the mean heterogeneity of the yeasts originating from the plus-Pb incubations remained greater than that of the control yeasts (GLMM: chi-squared = 8.741, df = 1, *P* = 0.003). There was no overall change in heterogeneity during the 25 generations (chi-squared = 1.924, df = 1, *P* = 0.164) and no change in the difference in heterogeneity between the two sets of four yeasts during this period (chi-squared = 1.072, df = 1, *P* = 0.301). Therefore, the relatively high heterogeneity that evolved in the presence of Pb during the 500-generation experiment was retained after 25 generations in the absence of Pb, implying that this phenotypic heterogeneity was a heritable trait.

## Discussion

It has been widely suggested that heterogeneity among individual cells of genetically uniform populations confers a survival advantage during environmental perturbation. This idea has been supported by computer simulations and laboratory studies with model organisms, including bacteria and the yeast *S. cerevisiae* (Thattai and van Oudenaarden, 2004; Blake *et al*., 2006; Smith *et al*., 2007; Gaal *et al*., 2010), but crucially has not previously been tested with wild microorganisms in natural habitats. The present study provided three key insights to this phenomenon as outlined below.

First, it was shown that phenotypic heterogeneity is prevalent in the wild yeasts that we studied: *C. podzolicus*, *C. sake* and *S. roseus.* These comprise both ascomycete and basidiomycete yeasts, suggesting that this phenomenon is likely to be widespread in the fungal taxa. *Cryptococcus podzolicus* is a frequently isolated soil yeast that may grow in the rhizosphere of a number of plant species; *C. sake* is found in diverse habitats, including plants (tree sap, vegetables) and in natural fermentations; *S. roseus* is one of the most common phylloplane yeasts in many temperate geographical locations worldwide (Kurtzman *et al*., 2010).

Second, we found that isolates with the highest phenotypic heterogeneities were consistently recovered from the various polluted environments visited during the study. By contrast, isolates with the lowest heterogeneities were found at matched control (unpolluted) locations. Consistent with these observations, laboratory growth tests demonstrated that isolates of *C. sake* with higher heterogeneity exhibited greater relative fitness in the presence of lead (the relevant environmental stressor) than isolates with lower heterogeneity. This indication that heterogeneity relates positively to survival in adverse conditions in the wild is a key finding. It provides evidence from the natural environment to support the hypothesis that phenotypic heterogeneity confers an advantage during environmental perturbation (Thattai and van Oudenaarden, 2004; Blake *et al*., 2006; Bishop *et al*., 2007; Smith *et al*., 2007; Acar *et al*., 2008; Gaal *et al*., 2010). It is also important because traditional measurements of mean (culture-averaged) resistance of an organism to a stressor, e.g. IC_50_, are widely used to indicate selection of resistant organisms in polluted environments (Bishop *et al*., 2007; Adamo *et al*., 2012). However, our results indicated that heterogeneity and IC_50_ values are not related, and that heterogeneity can also be a significant indicator of an organism's ability to persist in a polluted habitat.

Third, the laboratory evolution experiments with *C. sake* showed that increased heterogeneity is a trait that can be selected for during long-term environmental stress of wild microbial isolates. As mentioned above, there has been accumulating evidence with laboratory microorganisms of various genetic bases for phenotypic heterogeneity, for example through TATA box sequence changes in gene promoters (Raser and O'Shea, 2004; Blake *et al*., 2006; Newman *et al*., 2006; Freed *et al*., 2008; Li *et al*., 2010; Hornung *et al*., 2012; Silander *et al*., 2012; Carey *et al*., 2013). The finding from the present evolution experiments, in conjunction with a competitive advantage of isolates with higher pre-existing heterogeneity, implies that both mechanisms may account for the observed occupation of polluted environments by high-heterogeneity yeasts in the wild.

The advantages conferred by heterogeneity during stress appear to be counterbalanced by a fitness cost under standard growth conditions. In our assays, there was a small negative correlation between growth and heterogeneity when isolates were grown without stressors, consistent with findings elsewhere (Wang and Zhang, 2011). The extant level of phenotypic heterogeneity in a genetically uniform population is likely to be balanced between such costs (under standard conditions) and benefits (during stress). Accordingly, phenotypic heterogeneity can be considered a bet-hedging strategy (Beaumont *et al*., 2009; Levy *et al*., 2012). The long-term balance between costs and benefits of heterogeneity depends on the future instability of the habitat, with instability favouring the trait of heterogeneity. Consequently, environments subject to frequent perturbations will, our results indicate, select for organisms with increased heterogeneity. An alternative possibility could be that high heterogeneity is the common or default phenotype, with low heterogeneity evolving under constant non-stress conditions. However, our experimental evolution assays did show that increased heterogeneity evolved in wild isolates under stress in the laboratory. Quantitative empirical support for bet hedging has been judged to be lacking (Simons, 2009); the environmental selection of heterogeneity observed in the present study provides evidence that helps address this issue.

**Figure 7:**
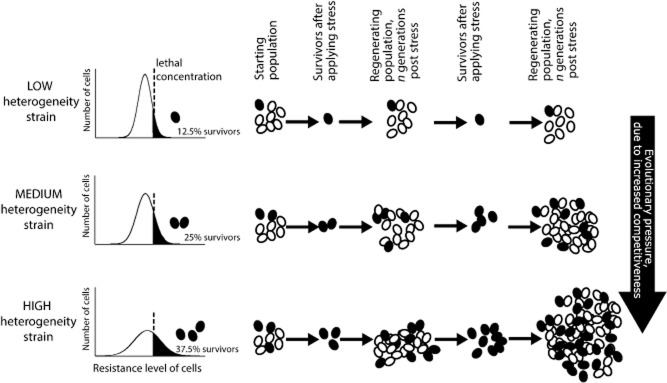
Schematic showing how the selective pressure of stress favours heterogeneous organisms. Three example strains are illustrated, with the same mean resistances to a given stress (approximating to the peaks in the histograms) but with different heterogeneities (reflected by histogram width). In the low-heterogeneity strain, 12.5% of cells fall above the survival threshold at the indicated stressor dose, whereas the medium- and high-heterogeneity strains comprise 25% and 37.5% survivors at the same dose respectively. For clarity in this example, each round of stress application is followed by a recovery period (of three generations) during which resistant survivors re-seed heterogeneous populations. The scheme illustrates how each successive round of stress followed by recovery amplifies the relative numbers of cells in the more heterogeneous populations. This faster outgrowth of the heterogeneous strains is the same process that will favour any higher heterogeneity mutants that may spontaneously arise in the populations, ultimately leading to increased heterogeneity that is heritably stable.

As depicted in Fig. 7, the slight fitness advantage of heterogeneous organisms during stress, observed in this study with Pb stress, would be expected to cause high-heterogeneity organisms to outcompete low-heterogeneity organisms over a number of cell generations. Previous laboratory studies have yielded similar conclusions, particularly where the stress is fluctuating or intermittent (Thattai and van Oudenaarden, 2004; Acar *et al*., 2008; Gaal *et al*., 2010). By comparison, the pollutants at the various environmental sites sampled here might be thought to give more constant exposure. However, there would be intermittent dilution by rainfall, in addition to changes in direction and intensity of the wind (affecting SO_2_ deposition). In conjunction with fluctuations in pollutant discharges at the mine or coking-plant sources visited here, there will inevitably have been fluctuations in the intensity of stress to which the native yeast isolates were exposed. Even in the case of our evolution assays, the stress exerted by the Pb supplement would be expected to change during the course of each batch culture because of changes in cell density and parameters that affect metal bioavailability, such as pH and dissolved O_2_ (Hughes and Poole, 1991; Gadd, 1993). Our study harnesses fluctuations in environmental stress that are not directly controlled by a researcher but that occur naturally. Accordingly, it provides a realistic portrayal of the impact of heterogeneity as it applies to populations under natural conditions. It should be noted that heterogeneity can also be expected to provide some advantage where an environment becomes subject to more constant environmental stress (Fig. 7). It seems less likely that selection for heterogeneity would be greater than for mean stress resistance under such constant conditions, although we cannot discount that the trait of heterogeneity may offer a larger mutational target in cells, i.e. it could be ‘easier’ to evolve heterogeneity.

As mentioned above, the present evidence suggests that outcompetition by heterogeneous organisms can at least partly explain the apparent real-life situation in which polluted habitats favour organisms with high heterogeneity (Fig. 7). The relative importance of outcompetition by organisms with pre-existing high heterogeneity, versus longer-term evolution of heterogeneity by mutation, may be especially great in transiently populated habitats; in such cases, like the deciduous phylloplane of Site 3, there will be a shorter window of opportunity for evolution to occur. A further observation is that the advantage of heterogeneity during laboratory growth under Pb stress was less marked with isolates from the unpolluted location than from the Pb-polluted location. Given that the pre-existing heterogeneity trait of each isolate was heritably stable, this suggests that condition (e.g. Pb-polluted versus unpolluted locations) may affect the nature of heterogeneity that evolves over time in the wild, with resultant heterogeneity phenotypes being ‘tuned’ to a relevant selective agent(s). It is also thought that there is tuning of the rate at which cells switch between phenotypes to the frequency of environmental change (Acar *et al*., 2008).

The wild yeast isolates from this study provide a novel and unique resource. Their diverse heterogeneities offer the opportunity to understand further how the environment can shape this important trait. At present, the molecular bases for inter-strain variation in cell-to-cell heterogeneity arising in natural ecosystems are not known. Existing knowledge of the genetic (i.e. evolvable) drivers of heterogeneity is based on laboratory studies with model organisms. Several heterogeneously expressed resistance genes that contribute significantly to the gradients of response plots to different stressors have been identified in *S. cerevisiae* (Sumner *et al*., 2012; Bishop *et al*., 2007; Smith *et al*., 2007). In these cases, deterministic parameters like cell cycle and age- or rhythm-dependent gene regulation were important for the heterogeneity. The occurrence of prion-like proteins in wild yeasts has suggested a further potential driver of heterogeneity, as prion-dependent mistranslation or transcriptional repression can generate cell diversity (Halfmann *et al*., 2012; Holmes *et al*., 2013). However, this seems unlikely to be important with the present yeast isolates because prion-dependent, single-cell phenotypes tend to be heritable, and curing guanidine hydrochloride-susceptible prions from our isolates did not alter their heterogeneities (A. Porquier, S. L. Holland and S. V. Avery, unpublished data).

Our results indicate that non-genotypic heterogeneity is an important trait for organisms in the natural environment, contributing to their competitiveness. Evolution and selection are key processes that appear to drive increased heterogeneity among yeasts experiencing adverse conditions. We infer that such cell-to-cell heterogeneity makes a key contribution to intra-species diversity (and associated fitness) of wild populations, which is complementary to that attributable to genotypic diversity. A recent report highlighted the negative impact of human activities on genotypic diversity in natural environments, with an increased risk of ecosystem collapse (MacDougall *et al*., 2013). According to the new findings of the present study, pollution arising from human activities has the opposite effect on non-genotypic heterogeneity, increasing the diversity among individual organisms. It is tempting to suggest that such responses might help buffer the reported impact of anthropogenic disturbances on genotypic diversity, helping sustain the integrity of natural ecosystems.

## Experimental procedures

### Study sites

Samples were collected from three sites between 2009 and 2011, each comprising a polluted location and nearby non-polluted (control) location. Records of pollutant levels were available for each location and site. Site 1 comprised a copper-polluted location at the edge of a pool at the abandoned Devon Great Consols mine complex, Devon, UK (UK Ordnance Survey: map coordinates SX426733 N50:32:52 W4:13:25) and a similar off-site control location ∼20 km north of the mine complex (UK Ordnance Survey: map coordinates SX418901 N50:68:69 W4:24:03) (Langdon *et al*., 2001; Kille *et al*., 2013). Site 2 comprised a lead-polluted location (UK Ordnance Survey: Map coordinates SN865938 N52:31:50 W3:40:22) and a corresponding control location ∼ 2 km upstream (UK Ordnance Survey: map coordinates SN853939 N52:31:51 W3:4:26). The two locations were downstream and upstream, respectively, of the effluent discharging from the Dylife mine in Wales, UK (Atkins, 2008). Site 3 comprised a sulphur dioxide-polluted location (UK Ordnance Survey: map coordinates. SE929411 N53:35:44 W00:35:49) and a nearby (∼ 2 km) control location (UK Ordnance Survey: map coordinates SE918087 N53:34:01 W00:36:54) close to the coking plant at Corus Steelworks, near Scunthorpe, UK (http://www.nlincs.aeat.com).

### Yeast sampling and identification

Sediment or soil samples were collected from Sites 1 and 2 in sterile 50 ml tubes. Within 6 h of sampling, the sediments were vortexed in sterile water and plated at different dilutions on MYP agar [malt extract (Sigma, St Louis, Missouri, USA) 7 gl^−1^, yeast extract (Oxoid, Cambridge, UK) 0.5 gl^−1^, soytone (BD Bacto, New Jersey, USA) 2.5 gl^−1^, agar (Sigma) 15 gl^−1^], supplemented with chloramphenicol (100 mgl^−1^) (Holland *et al*., 2011). At Site 3, leaves were picked from a variety of tree species and transported aseptically to the laboratory. Leaves were either pressed directly to MYP, agar as outlined previously (Inacio *et al*., 2005), or vortexed with sterile water before spread-plating wash samples to MYP agar. After incubation at room temperature for 4 days, individual yeast colonies were subcultured onto fresh MYP agar to enable further characterization.

For identification purposes, the internal transcribed spacer regions (regions 1 and 2, and the intervening 5.8S rDNA sequence) were amplified from isolates by polymerase chain reaction (PCR) and digested, as described previously (Esteve-Zarzoso *et al*., 1999). Digestion products were subsequently compared, enabling similar species to be grouped prior to purification of PCR products by phenol/chloroform extraction and ethanol precipitation and sequencing of representative isolates. Sequencing was carried out as described previously (Holland *et al*., 2011), and sequence data were compared against existing databases with the blast programme (http://www.ncbi.nlm.nih.gov/BLAST/) at the National Centre for Biotechnology Information, and the Wu-Blast programme at http://www.ebi.ac.uk/Tools/sss/wublast/. Next, DNA was extracted from individual isolates (Hoffman and Winston, 1987), and RAPD-PCR fingerprinting was performed to assess clonality of isolates, using primers OPW08, OPW09, OPAX2, OPW04 (Site 1); OPAJ03, OPW05, OPW06 (Site 2); or OPAJ01, OPAJ03, OPA05, OPAX20, OPW10 (Site 3), as described previously (O'Gorman *et al*., 2009).

### Determination of heterogeneity and IC_50_ values

Yeasts were cultured overnight in MYP broth, then subcultured to fresh medium and incubated for a further 6 h. Cells were harvested by centrifugation and suspended to ∼ 3000 cells ml^−1^ in phosphate buffered saline (PBS), before spread plating ∼ 200 colony forming units (CFUs) to MYP agar supplemented with stressors, as specified. CFUs were enumerated after 14 days of incubation at room temperature. Experiments were repeated on three independent days, with plating in triplicate for each isolate and condition on each day. Percentage viability within each experiment was determined with reference to mean CFUs on control (minus stressor) plates.

To model the effect of stressor concentration (*x*) on viability (*y*), a three-parameter version of the Weibull survival equation was applied. This was a modified version of the four-parameter Weibull equation (Crawley, 2007): *y* = *a* − *b*exp(−*cx^d^*), where *a* is the upper asymptote of the survival curve, *b* is the drop in viability between the upper asymptote and the *y* intercept, *c* is a rate constant, and *d* alters the steepness of the central part of the curve from a shallow s-shape at low values to a steep step-function at high values. We modified this function by reflecting about the *y* axis, such that maximum viability occurs at *x* = 0. We also set *b* = *a* to allow the asymptote to vary while ensuring that zero viability is reached at high stressor concentrations. For each isolate on each independent day of analysis in each experiment, three parameters describing the model fit (*a*, *c* and *d*) were extracted, and the IC_50_ calculated as the concentration of the stressor at which viability was 50%. The Weibull equation for each isolate on each day was then differentiated to find the slope of the curve [a measure of heterogeneity (Sumner *et al*., 2012; Bishop *et al*., 2007)] at the IC_50_ of the stressor. Note that the s-shaped survival curves (Fig. 1) result from differences in the concentrations of environmental stressor that are sufficient to kill individual cells, which in turn are caused by continuous variation among cells in a hypothetical resistance trait. The shape of the distribution of this trait will determine the exact shape of the survival curve. For example, if the trait is normally distributed, the resultant survival curve will be equivalent to the cumulative probability density function of the normal distribution, with a slope that is determined by the trait's standard deviation. For our data, in the absence of any specific information about the shape of the underlying trait distribution, we fitted the most parsimonious mathematical function to the survival data and calculated the gradient of this function at IC_50_ as a direct proxy for the variance (i.e. heterogeneity). These gradients and IC_50_ values were compared for yeast isolates from polluted versus unpolluted locations at each of the three sites, using a linear mixed-effects model with type of location (polluted versus unpolluted) as a fixed factor and isolate as a random factor (to account for the fact that each isolate was tested on more than one day). The tests were one-tailed as the *a priori* hypotheses were that isolates from polluted locations would have more heterogeneous resistance to the stressor as well as higher IC_50_ than those from unpolluted locations. All analyses were conducted in R version 2.15.0 (R-Core-Team, 2013). Weibull models were fitted using the nls (non-linear least squares regression) package.

### Growth and evolution experiments

To compare growth in broth culture during exposure to lead as a representative stressor, isolates with different heterogeneities from Site 2 were cultured to exponential phase, as described above. Cells were then subcultured to fresh MYP broth, either supplemented or not with 15 μM Pb(NO_3_)_2_, and incubated with shaking in a BioTek Powerwave XS microplate spectrophotometer (Winooski, Vermont, USA) as described previously (Alhebshi *et al*., 2012). After 24 h, cells were subcultured to the same fresh medium with subsequent incubation for a further 24 h, and this was repeated to give a final growth duration of 3 days. Final OD_600_ values after 3 days of incubation in the presence or absence of Pb were used to assess relative growth.

For evolution experiments, isolates isolated from the unpolluted location at Site 2 were subcultured daily to MYP broth supplemented with 0 or 30 μM Pb(NO_3_)_2_ and incubated as above for a total of 60 days, representing ≥ 500 generations (Ferea *et al*., 1999; Dunham *et al*., 2002). Heterogeneity in the Pb resistance of each culture was re-assayed, as described above, after the ≥ 500 generation incubation. The heritability of heterogeneity in evolved cultures was assayed after incubation in the absence of Pb for ∼25 generations before retesting Pb resistance.

## Supporting Information

**Fig. S1.** Sample data illustrating non-heritability of individual-cell stress resistance of wild yeast isolates. The examples are *C. sake* isolates collected near a lead-mine outflow (i.e. Site 2, which gave the least significant effect of stressor on selection for heterogeneity). Resistant colonies of each isolate that grew at 1.5 mM Pb(NO_3_)_2_ were picked then grown in the absence of Pb, before re-assaying resistance at 1.5 mM Pb(NO_3_)_2_. The figures shows percentage colony formation in this re-assay of cultures originating from the ‘resistant’ colonies (▪), versus the same assay for naïve cultures of the isolates, not previously selected by Pb-resistance (□).

**Fig. S2.** Heterogeneity and IC_50_ are not correlated. Scatter plots are presented for heterogeneity versus IC_50_ values of every isolate investigated from the six locations. The *P*-values presented are for correlations across the isolates from each location.
