# Supplementary figures and images for: Phenotypic heterogeneity is a selected trait in natural yeast populations subject to environmental stress
Source: Environ Microbiol. 2013 Sep 3;16(6):1729–40. doi: 10.1111/1462-2920.12243 (PMC4231229; doi:10.1111/1462-2920.12243)

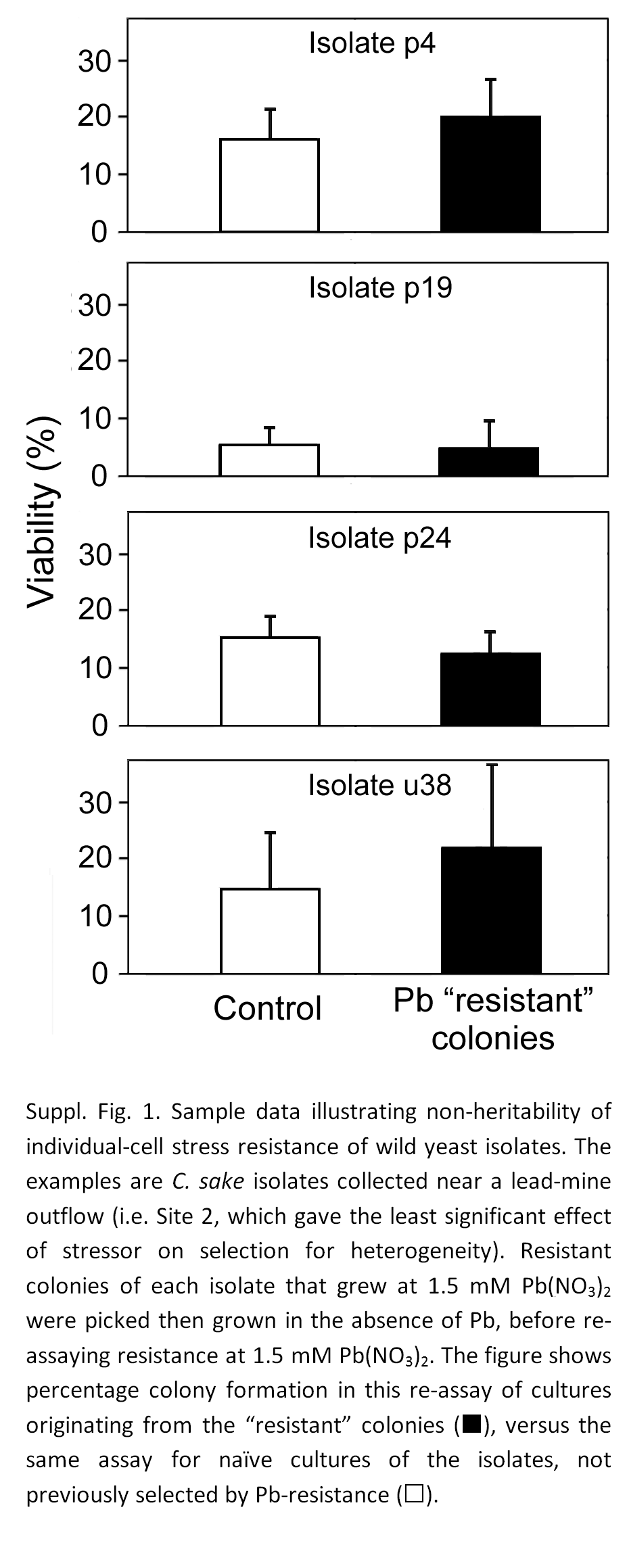

Supplement: Supplementary file 1 — Fig. S1. Sample data illustrating non-heritability of individual-cell stress resistance of wild yeast isolates. The examples are C. sake isolates collected near a lead-mine outflow (i.e. Site 2, which gave the least significant effect of stressor on selection for heterogeneity). Resistant colonies of each isolate that grew at 1.5 mM Pb(NO3)2 were picked then grown in the absence of Pb, before re-assaying resistance at 1.5 mM Pb(NO3)2. The figures shows percentage colony formation in this re-assay of cultures originating from the ‘resistant’ colonies (▪), versus the same assay for naïve cultures of the isolates, not previously selected by Pb-resistance (□). [file emi0016-1729-sd1.tif]

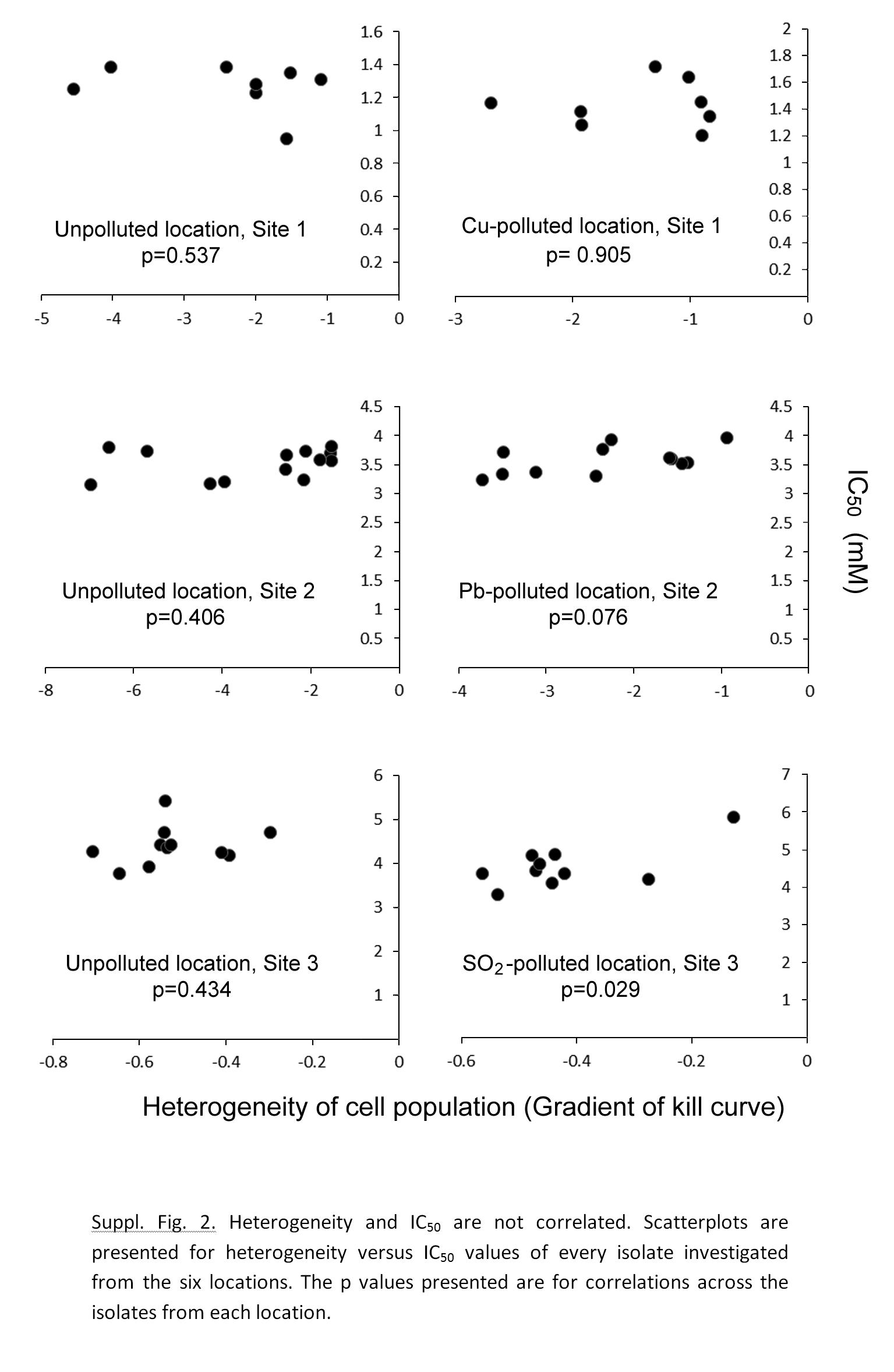

Supplement: Supplementary file 2 — Fig. S2. Heterogeneity and IC50 are not correlated. Scatter plots are presented for heterogeneity versus IC50 values of every isolate investigated from the six locations. The P-values presented are for correlations across the isolates from each location. [file emi0016-1729-sd2.tif]
